# Potentially inappropriate medications prescribing to elderly patients with advanced chronic kidney by using 2019 American Geriatrics Society Beers Criteria

**DOI:** 10.1002/hsr2.214

**Published:** 2020-12-07

**Authors:** Bahia Chahine

**Affiliations:** ^1^ School of Pharmacy Lebanese International University Beirut Lebanon

**Keywords:** Beers criteria, chronic kidney disease, hemodialysis, potentially inappropriate medication

## Abstract

**Background and aims:**

A potentially inappropriate medication (PIM) is defined as a drug‐carrying risks outweighing the expected clinical benefits. Elderly patients with chronic kidney disease (CKD) are particularly at higher risk of drug‐related toxicities. In Lebanon, no studies have been conducted regarding the prescribing of PIMs in hospitalized CKD patients. This study aimed to check the prevalence of PIMs using the American Geriatrics Society (AGS) Beers criteria in elderly patients with advanced CKD stages including dialysis and to identify possible risk factors that may be associated with prescribing PIMs in this population.

**Methods:**

A retrospective cross‐sectional study was conducted on patients with advanced CKD above the age of 65 years and admitted between January 2019 and June 2019 to two University Hospitals in Beirut, Lebanon. We used multiple logistic regression analysis to determine which factors were associated with prescription of PIMs according to AGS Beers criteria‐2019.

**Results:**

The study sample included 199 patients with renal dysfunction, 75.9% were aged 70 years or more, 53.8% were females, and 61.8% were prescribed five drugs or more. Eighty‐two patients were receiving hemodialysis (41.2%). PIMs prevalence was 34.1% (68/199 patients) according to Beers criteria in elderly patients with advanced CKD stages.

The most frequently prescribed PIMs were ranitidine (39.1%), enoxaparin (25%), tramadol (9.8%), and ciprofloxacin (5.4%). Polypharmacy (OR 2.1, CI 95% 1.58‐2.79), a higher number of comorbidities (OR 3.01, CI 95% 1.43‐6.30), and coronary artery diseases (OR 3.14 CI 95% 1.44‐6.85) were the factors associated with an increased risk of at least one PIM prescription.

**Conclusion:**

Our study found that one out of three patients with advanced CKD had at least one PIM according to the latest Beers criteria. A large proportion of inappropriate prescribing is preventable by increasing awareness of prescribing physicians to the explicit lists of PIMs.

## 1 INTRODUCTION

Chronic kidney disease (CKD) is a substantial public health problem with a global prevalence of 9.1%.^1^ It is increasingly recognized as a crucial comorbid condition in elderly individuals aged >65 years.^2^ Older adults with renal impairment are at high risk of drug‐related problems due to their multiple comorbidities, polypharmacy, and the alterations in pharmacokinetics and pharmacodynamics of kidney excreted drugs due to reduced filtration rate, renal metabolism, or impaired tubular function.^3^

The burden of polypharmacy, typically defined as using five or more medications per day, is high in this population. In CKD stages 2 to 5, patients have been prescribed a median of eight different medications and increases to 10 to 12 medications in dialysis patients.^4^, ^5^

Polypharmacy increases the risk of prescribing potentially inappropriate medications (PIMs), consequently increasing adverse effects.^6^ PIMs are usually defined as “medications that should be avoided due to their risk which outweighs their benefit and when there are equally or more effective but lower risk alternatives are available.”^7^

Lately, various tools have been created to evaluate the appropriateness of medication use in the elderly.^8^ The Beers Criteria are among the most widely used, it was first published almost 30 years ago, making them longtime used criteria by health care providers. This tool is primarily intended for practicing clinicians to manage and improve prescribing practices in older adults.^9^

Optimal application of the Beers Criteria involves recognizing PIMs and offering safer alternatives where applicable. Clinicians can use this list when reviewing medications for a new patient or prescribing new medications regardless of whether they are hospitalized, institutionalized, or ambulatory.^9^ There are two version of the American Geriatrics Society (AGS) Beers criteria focused on kidney function (2015 and 2019),^10^, ^11^ the most recent update was released in 2019 and contains an updated list of 23 medications that should be avoided or have their dosage reduced based on kidney function.^11^ Two antibiotics have been added, ciprofloxacin and TMP‐SMX. Dofetilide was also added and the creatinine clearance lower limit at which to avoid edoxaban has been reduced to less than 15 mL/min.^11^

PIMs prescribing is considered one of the commonly experienced drug‐related issues and its current rates in elderly CKD patients is alarming. A study of elderly hospitalized CKD patients found that 48% of patients were prescribed at least one PIM.^8^, ^9^, ^10^, ^11^, ^12^ So far, the estimated prevalence of PIMs varied from 36% to 56% in patients with eGFR <30 mL/min/1.73m^2^ using Beers Criteria.^13^, ^14^ Moreover, in elderly dialysis patients, the reported prevalence of PIMs ranged from 43% to 57%.^15^, ^16^

Polypharmacy and the presence of comorbid conditions were the biggest factors associated with increased risk of prescribing PIM among elderly patients.^17^ Several studies have shown that PIMs affect patients' morbidity, emergency department visits, hospitalization, mortality rates, and the adverse effects of PIMs add significantly to healthcare utilization and cost.^18^, ^19^, ^20^ There is evidence that adverse drug reactions could be prevented by avoiding prescribing errors,^21^ therefore, early detection of PIM may improve the quality of treatment.

In Lebanon, no studies have been conducted regarding PIMs prescription in hospitalized CKD patients. The study aimed to check the prevalence of PIMs using the AGS Beers criteria in elderly patients with advanced CKD stages including dialysis and to identify possible risk factors that may be associated with prescribing PIMs in this population.

## 2 METHODS

### 2.1 Study design, setting, and participants

This retrospective cross‐sectional study was conducted between January 2019 and June 2019 at two university hospitals in Beirut, Lebanon. The study inclusion criteria were as follows: all elderly patients aged ≥65 years admitted to the hospital for ≥ one night, received at least one medication, and diagnosed with advanced CKD stages G4 (GFR 15‐29 mL/min) or G5 (GFR <15 mL/min).^22^ Those who had an incomplete medical record were excluded. The study protocol was reviewed and approved by the Institutional Review Board of each hospital. The anonymity and confidentiality of patients and their data were protected at all times.

### 2.2 Data collection and variables

Databases of the two hospitals were computerized; medical records of all patients admitted during the study period to any hospital ward were screened. Initially, databases were searched using the International Statistical Classification of Diseases and Related Health Problems (ICD‐10)^23^ coding system for code of N18 (CKD).

Two independent clinical pharmacists gathered data from digital medical records about patient demographics, medications, diagnoses, comorbidities, and admissions through Open Data Kit (ODK) collect android application, and directly uploaded to Microsoft excel 2019. The Charlson Comorbidity Index (CCI) was used to quantify the presence of co‐existing diseases using an online calculator.^24^ Patients' reason of admission was recorded as renal if it was CKD or its complications, uremic symptoms, or dialysis. Nonrenal reasons of admission included all other admission types such as cardiac diseases, infections where a nephrologist might not be consulted. For this study, all drugs listed on the patient record during hospitalization were evaluated for PIM. The medication was considered as PIM if it is found in the sixth table of the 2019 Beers list of medications that should be avoided or its dose should be reduced with advanced stages of kidney dysfunction in older adults (Table S1).

Independent study variables were gender, age range, number of drugs prescribed, CCI, CKD stage, the reason for admission, dialysis, ward (Cardiology Unit, Internal Medicine, Intensive Care Unit) and length of hospitalization. The dependent variable was the number of patients with at least one PIM detected in their medication list in the hospital.

### 2.3 Statistical analysis

Data were collected and analyzed by Statistical Package for the Social Sciences SPSS, Version 23. Frequencies and percentages were used for categorical variables, whereas means and standard deviations (SD) for continuous variables. The prevalence of PIM among CKD patients was calculated by dividing the number of elderly patients with advanced CKD who received at least one PIM by the total number of elderly patients with advanced CKD. Univariate analysis was performed to check for associations between independent variables and the prevalence of PIM. Adjusted odds ratios with their 95% confidence intervals (CI) were estimated by logistic regression analysis to determine which factors were associated with prescription of PIMs. *P* value < .05 was considered statistically significant.

## 3 RESULTS

The study sample included 199 patients with renal dysfunction, 75.9% of patients were aged 70 years or more and 53.8% were females. The majority of subjects (88.9%) were admitted for non‐renal reasons such as infections and cardiac complaints and the median length of hospitalization was 8 days. Eighty‐two patients were receiving hemodialysis (41.2%). Hypertension was the most common comorbidity affecting 94% of subjects followed by, coronary heart disease and diabetes mellitus 72.4% and 55.8%, respectively. Systemic infections were found in 48.2% of patients and chronic obstructive pulmonary disease in 19.6%. Other less frequent comorbidities were cancer (16.1%) and liver diseases (2.5%). More than half of the subject had a CCI between 5 and 9. The median number of medications taken per patient was 7% and 61.8% of subjects were prescribed five drugs or more. Table 1 displays the detailed patient characteristics.

**TABLE 1:** Characteristics of the study population

Variable	Frequency (%) (n = 199)	Mean (SD)	Patients with at least one PIM medication (%) (n = 68)	Univariate analysis *P* value
Gender				
Male	92 (46.2)		28 (30.4)	.91
Female	107 (53.8)		40 (37.4)	
Age (year)				
65‐69	48 (24.1)	76.47 (8.2)	16 (33.3)	.71
≥70	151 (75.9)		52 (34.4)	
Reason for admission				
Renal	22 (11.1)		6 (27.3)	.43
Non‐renal	177 (88.9)		62 (35)	
Hospital ward				
IM	75 (37.7)		31 (41.3)	.59
CCU	44 (22.1)		16 (36.4)	
ICU	56 (28.1)		15 (26.8)	
Other	24 (12.1)		6 (25)	
Length of hospitalization				
≤7 days	87 (43.7)	8.7 (2.8)	28 (32.2)	.65
≥8 days	112 (56.3)		40 (35.7)	
Median (range)		8 (3‐40)		
CKD stage				
G4	101 (50.8)		31 (30.7)	.42
G5	98 (49.2)		37 (37.8)	
Dialysis				
Yes	82 (41.2)		33 (40.2)	.27
No	117 (58.8)		35 (30)	
Hypertension	187 (94)		67 (35.8)	.55
Coronary heart disease	144 (72.4)		43 (29.9)	.04
Diabetes mellitus	111 (55.8)		32 (28.8)	.02
Systemic infections	96 (48.2)		8 (8.3)	.58
Chronic obstructive pulmonary disease	39 (19.6)		3 (7.7)	.72
Cancer	32 (16.1)		0	‐
Liver diseases	5 (2.5)		0	‐
Charlson Comorbidity Index				
2–4	95 (47.7)	6.5 (2.4)	25 (26.3)	.03
5–9	104 (52.3)		43 (41.3)	
Number of drugs				
<5	76 (38.2)	5.98 (1.3)	11 (14.5)	.00
5–10	123 (61.8)		57 (46.3)	
Median (range)		7 (3‐14)		

Abbreviations: CCU, cardiac care unit; CKD, chronic kidney disease; ICU, intensive care unit; IM: internal medicine.

**FIGURE 1:**
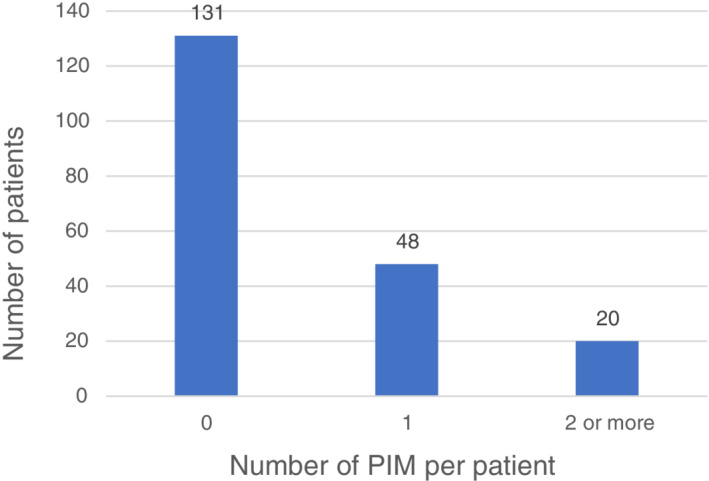
Number of potentially inappropriate medications per patient according to 2019 American Geriatrics Society Beers criteria

Sixty‐eight patients were found to have at least one PIM according to Beers criteria with a prevalence of 34.1%. Of these, 48 patients had one PIM and 20 patients had two or more PIMs (Figure 1).

In dialysis patients, 33 out of 82 had at least one PIM making the prevalence of PIMs 40.2% vs 30% in nondialysis patients.

Univariate analysis identified statistically significant differences in PIM prescription among subjects with respect to the number of drugs prescribed (*P* = .00), the number of comorbidities (*P* = .03), and specific diseases as diabetes mellitus (*P* = .02) and coronary heart diseases (*P* = .04). At least one PIM was found to be more common in females, internal medicine service, CKD G5 stage and hospitalized for 8 days or more but there were no significant differences.

Twelve out of the 23 (52%) medications listed in the sixth table of explicit AGS Beers criteria for CKD were detected in our sample population, while the remaining 11 drugs were not encountered. In total, 1191 drugs were prescribed, 92 PIMs were identified using the AGS Beers criteria. The most frequently prescribed PIMs to older CKD patients included ranitidine (39.1%) and enoxaparin (25%). Besides, tramadol and pregabalin accounted for about 20% of identified PIMs. Less frequently encountered PIMs were ciprofloxacin, fondaparinux, apixaban, dabigatran, gabapentin, and levetiracetam. Table 2 summarizes the drugs implicated in PIM prescriptions.

**TABLE 2:** Prescribed PIMs according to Beers criteria^†^

Drug	Criteria	Overall use frequency (%) n = 1191	PIM use frequency (%) n = 92
Gastrointestinal		138 (11.6)	36 (39.1)
Ranitidine	Reduce dose	138 (11.6)	36 (39.1)
*Cardiovascular or hemostasis*		88 (7.4)	32 (34.8)
Enoxaparin	Reduce dose	79 (6.6)	23 (25)
Fonadaparinux	Avoid	4 (0.3)	4 (4.3)
Spironolactone	Avoid	2 (0.17)	2 (2.2)
Apixaban	Avoid	1 (0.084)	1 (1.1)
Dabigatran	Avoid	1 (0.084)	1 (1.1)
Rivaroxaban	Avoid	1 (0.084)	1 (1.1)
*Central nervous system and analgesics*		133 (11.1)	19 (20.7)
Tramadol	Immediate release: reduce dose	67 (5.6)	9 (9.8)
Pregabalin	Reduce dose	46 (3.8)	8 (8.7)
Gabapentin	Reduce dose	14 (1.2)	1 (1.1)
Levetiracetam	Reduce dose	6 (0.5)	1 (1.1)
*Anti‐infective*		19 (1.6)	5 (5.4)
Ciprofloxacin	Reduce dose	19 (1.6)	5 (5.4)

^†^ Eleven drugs in the sixth table of Beers criteria (Trimethoprim/sulfamethoxazole, amiloride, dofetilide, edoxaban, triamterene, duloxetine, cimetidine, famotidine, nizatidine, colchicine, and probenecid) were not detected in our sample.

Abbreviation: PIM, potentially inappropriate medication.

Table 3 displays the results of the logistic regression analysis. The higher number of prescribed drugs (OR 2.1, CI 95% 1.58‐2.79), the higher number of comorbidities (OR 3.01, CI 95% 1.43‐4.30), and coronary artery disease (OR 3.14 CI 95% 1.44‐4.85) were the factors associated with an increased risk of using at least one PIM. The logistic regression analysis of the remaining variables did not reveal any significant differences in the number of PIM identified by the Beers criteria.

**TABLE 3:** Logistic regression analysis

	*P*‐value	AOR	95% Confidence interval	
			Lower	Upper
Gender	.467	0.761	0.366	1.586
Age	.475	0.984	0.940	1.029
Reason for admission	.614	0.742	0.232	2.372
Hospital ward				
IM	.393			
CCU	.308	2.008	0.526	7.662
ICU	.410	1.788	0.449	7.121
Other	.982	0.984	0.243	3.983
Length of hospitalization	.260	1.072	0.950	1.210
CKD stage	.218	0.583	0.247	1.376
Dialysis	.229	1.747	0.704	4.334
Number of drugs	.000 †	2.102	1.580	2.797
Charlson comorbidity index	0.004†	3.010	1.436	4.308
Diabetes mellitus	.093	1.803	0.907	3.586
Hypertension	.154	0.183	0.018	1.886
Coronary artery disease	.004†	3.142	1.441	4.851

Abbreviations: AOR, adjusted odds ratio; CCU, cardiac care unit; CKD, chronic kidney disease; ICU, intensive care unit; IM: internal medicine.

^†^ Significant.

## 4 DISCUSSION

As far as we know, this is the first study to estimate the prevalence of PIMs in Lebanese hospitalized older adults with renal impairment.

Our study indicates that the overall prevalence of PIMs in patients with chronic and end‐stage kidney disease was 34.1%. In patients with advanced CKD stages receiving conservative treatment, the prevalence of PIMs was 30% vs 40.2% in hemodialysis patients.

These findings were consistent with previously published data using Beers criteria.^12^, ^13^, ^14^, ^15^, ^16^ In a study conducted by Kondo et al,^16^ PIMs were identified in more than 50% of the dialysis patients. Hannah et al,^25^ found that 32.3% of hospitalized patients with renal impairment had PIMs. Whereas, the lowest frequency of PIMs (13%) in elderly patients with CKD stages 3 to 5 has been revealed by Jones and Bhandari.^14^ Our results were within the prevalence range (13%‐50%) reported by previous studies and the conceivable explanation behind the discrepancies in the estimated prevalence of PIMs may be the different prescribing practices of each country and research methodologies.

Overall, the histamine‐2 receptor blocker ranitidine was the most common medication identified as PIM in our study. These findings are consistent with Kondo et al,^16^ and Alhawassi et al.^26^ The main reason for this inappropriateness was using ranitidine without dose adjustment‐reduction that is, 5 mg IV every 6 hours or as 6.25 mg/hour continuous infusion, in patients with advanced CKD stages. Renal function impairment is associated with increased plasma ranitidine levels and altered disposition of the drug, which may result in central nervous system‐ adverse drug reactions such as lethargy, confusion, somnolence, disorientation, and possibly hallucinations.^27^

In Lebanon, ranitidine is commonly over‐prescribed for stress ulcer prophylaxis in hospitalized patients.^28^ Given this high frequency of ranitidine prescription, it is important to ensure appropriate dosing to minimize the risk of mental status change.^11^

Enoxaparin was identified in 25% of PIMs in our study. Similar results were obtained by Munish et al,^29^ 27% of patients who were overdosed based on of creatinine clearance (CrCl) had errors involved with enoxaparin. In all these patients, a 40 mg subcutaneous daily dose of enoxaparin was ordered while the correct dose had to be 30 mg daily as all the patients had CrCl <30 mL/min. In a nonrandomized, retrospective study, bleeding was significantly more common with enoxaparin among patients with severe renal insufficiency.^30^

Ishida and colleagues have also found that 43% of gabapentin and 45% of pregabalin prescriptions were inappropriate in dialysis cohorts,^31^ our results showed much lower rates because we studied both dialysis and non‐dialysis patients. According to Beers criteria, both drugs increase the risk of central nervous system adverse effects if used at normal doses in advanced CKD stages due to reduced clearance of these agents. In one study, analgesics attributed for 11% of PIMs in patients with CKD.^14^ These finding were consistent with ours, tramadol accounted for 9.8% of PIMs. Its use was associated with all‐cause mortality, altered mental status, hospitalization, and increased fracture risk in patients with kidney dysfunction.^32^, ^33^

Furthermore, conversely to our findings, benzodiazepines, first‐generation antihistamines, and αblockers were reported as the most common PIM classes in other studies using different criteria.^15^, ^16^

The majority of published data indicated that aging, late‐stage kidney disease, the high number of medications, and the presence of multiple comorbidities were major factors significantly associated with PIMs.^25^, ^34^, ^35^ In our study, only polypharmacy, multimorbidity, and coronary artery disease were associated with PIMs. There is clear evidence that the use of multiple medications to treat multiple comorbidities increase the risk of receiving inappropriate drugs.

In accordance with findings of San‐José et al,^36^ PIMs prevalence in our study was not affected by gender, the reason of admission, or length of hospitalization. The major causes accounting for the variability of risk factors associated with PIM prescribing include the study setting and country, patient characteristics, and the tools used for PIM detection.

This study is unprecedented in Lebanon to evaluate the pattern of PIM prescription in elderly patients with both chronic and end‐stage kidney disease receiving hemodialysis. Notwithstanding this, it had some limitations. First, our study was limited to two university hospitals in Lebanon, which may not reflect practices nationwide. Second, patients with incomplete medical records were excluded which could affect the results. Third, we evaluated the patterns of prescribing PIMs, but we did not investigate other medication‐prescribing problems as possible interactions between drugs, drug duplications, under‐prescribing, or prescribing omissions. Fourth, the correlation between PIMs and clinical outcomes were not assessed in our study.

## 5 CONCLUSION

Our study found that one out of three patients with advanced CKD had at least one PIM according to 2019 AGS Beers criteria. This high frequency of inappropriate prescribing is preventable by increasing awareness of prescribing physicians to the explicit lists of PIMs that are typically avoided or require dosing reduction based on renal function. Moreover, clinical pharmacist medication review can draw attention to PIMs through medication therapy management to achieve optimal prescribing practices in older adults.

## CONFLICT OF INTEREST

The author declares no conflict of interest.

## AUTHOR CONTRIBUTIONS

Methodology: Bahia Chahine

Writing ‐ Original Draft preparation: Bahia Chahine

Writing ‐ Review and Editing: Bahia Chahine

  All authors have read and approved the final version of the manuscript.

  Bahia Chahine had full access to all of the data in this study and takes complete responsibility for the integrity of the data and the accuracy of the data analysis.

### TRANSPARENCY STATEMENT

Bahia Chahine affirms that this manuscript is an honest, accurate, and transparent account of the study being reported; that no important aspects of the study have been omitted; and that any discrepancies from the study as planned (and, if relevant, registered) have been explained.

## Supporting information


**Table S1** Sixth table of the AGS Beers Criteria 2019

## Data Availability

The datasets used and analyzed during the current study are available on reasonable request.

## References

[1] GBD Chronic Kidney Disease Collaboration . Global, regional, and national burden of chronic kidney disease, 1990‐2017: a systematic analysis for the Global Burden of Disease Study 2017. Lancet. 2020;395(10225):709‐733. 10.1016/S0140-6736(20)30045-3. 32061315 PMC7049905

[2] Collins AJ , Foley RN , Gilbertson DT , Chen SC . United States Renal Data System public health surveillance of chronic kidney disease and end‐stage renal disease. Kidney Int Suppl (2011). 2015;5(1):2‐7. 10.1038/kisup.2015.2. 26097778 PMC4455192

[3] Mangoni A , Jackson S . Age‐related changes in pharmacokinetics and pharmacodynamics: basic principles and practical applications. Br J Clin Pharmacol. 2003;57(1):6‐14. 10.1046/j.1365-2125.2003.02007.x PMC1884408 14678335

[4] Laville SM , Metzger M , Stengel B , et al. Evaluation of the adequacy of drug prescriptions in patients with chronic kidney disease: results from the CKD‐REIN cohort. Br J Clin Pharmacol. 2018;84(12):2811‐2823. 10.1111/bcp.13738. 30110711 PMC6255993

[5] Peter WLS . Management of polypharmacy in dialysis patients. Semin Dial. 2015;28(4):427‐432. 10.1111/sdi.12377. 25864928

[6] Steinman MA , Landefeld CS , Rosenthal GE , et al. Polypharmacy and prescribing quality in older people. J Am Geriatr Soc. 2006;54:1516‐1523. 17038068 10.1111/j.1532-5415.2006.00889.x

[7] Page RL , II SA , Bryant LL , Ruscin JM . Inappropriate prescribing in the hospitalized elderly patient: defining the problem, evaluation tools, and possible solutions. Clin Interv Aging. 2010;5:75‐87. 10.2147/cia.s9564. 20396637 PMC2854054

[8] American geriatrics society identifies five things that healthcare providers and patients should question. J Am Geriatr Soc. 2013;61(4):622‐631. 10.1111/jgs.12226. 23469880 PMC3786213

[9] Ladda MA , Goralski KB . The effects of CKD on cytochrome P450‐mediateddrug metabolism. Adv Chronic Kidney Dis. 2016;23(2):67‐75. 10.1053/j.ackd.2015.10.002.6. 26979145

[10] American Geriatrics Society Beers Criteria Update Expert Panel . American Geriatrics Society 2015 updated Beers Criteria for potentially inappropriate medication use in older adults. J Am Geriatr Soc. 2015;63(11):2227‐2246. 26446832 10.1111/jgs.13702

[11] American geriatrics society 2019 updated AGS Beers Criteria® for potentially inappropriate medication use in older adults. J Am Geriatr Soc. 2019;67:674‐694. 10.1111/jgs.15767. 30693946

[12] Tesfaye WH , Wimmer BC , Peterson GM , et al. The effect of hospitalization on potentially inappropriate medication use in older adults with chronic kidney disease. Curr Med Res Opin. 2019;35(6):1119‐1126. 10.1080/03007995.2018.1560193. 30557066

[13] Secora A , Alexander G , Ballew S , Coresh J , Grams M . Kidney function, polypharmacy, and potentially inappropriate medication use in a community‐based cohort of older adults. Value Health. 2018;21:S266 10.1016/j.jval.2018.04.1795. PMC6093216 30039344

[14] Jones SA , Bhandari S . The prevalence of potentially inappropriate medication prescribing in elderly patients with chronic kidney disease. Postgrad Med J. 2013;89:247‐250. 23417370 10.1136/postgradmedj-2012-130889

[15] Parker K , Aasebø W , Stavem K . Potentially inappropriate medications in elderly haemodialysis patients using the STOPP criteria. Drugs. 2016;3(3):359‐363. 10.1007/s40801-016-0088-z. PMC5042944 27747833

[16] Kondo N , Nakamura F , Yamazaki S , et al. Prescription of potentially inappropriate medications to elderly hemodialysis patients: prevalence and predictors. Nephrol Dial Transpl. 2014;30(3):498‐505. 10.1093/ndt/gfu070. 24777993

[17] Alhawassi TM , Alatawi W , Alwhaibi M . Prevalence of potentially inappropriate medications use among older adults and risk factors using the 2015 American Geriatrics Society Beers criteria. BMC Geriatr. 2019;19(1):154 10.1186/s12877-019-1168-1. 31142286 PMC6542098

[18] Budnitz DS , Lovegrove MC , Shehab N . Emergency hospitalizations for adverse drug events in older Americans. N Engl J Med. 2011;365:2002‐2012. 22111719 10.1056/NEJMsa1103053

[19] Stockl KM , Le L , Zhang S , et al. Clinical and economic outcomes associated with potentially inappropriate prescribing in the elderly. Am J Manag Care. 2010;16:e1‐e10. 20059286

[20] Bradley MC , Fahey T , Cahir C , et al. Potentially inappropriate prescribing and cost outcomes for older people: a cross‐sectional study using the Northern Ireland Enhanced Prescribing Database. Eur J Clin Pharmacol. 2012;68(10):1425‐1433. 10.1007/s00228-012-1249-y. 22447297

[21] Tosato M , Landi F , Martone AM , et al. Potentially inappropriate drug use among hospitalised older adults: results from the CRIME study. Age Ageing. 2014;43:767‐773. 24637848 10.1093/ageing/afu029

[22] Kidney Disease: Improving Global Outcomes (KDIGO) CKD Work Group . KDIGO clinical practice guideline for the evaluation and management of chronic kidney disease. Kidney Int Suppl. 2013;3:1‐150.

[23] World Health Organization . International Classification of Diseases and Related Problems. 2020 https://icd.who.int/browse10/2019/en

[24] Hall WH , Ramachandran R , Narayan S , Jani AB , Vijayakumar S . An electronic application for rapidly calculating Charlson comorbidity score. BMC Cancer. 2004;4:94 10.1186/1471-2407-4-94. 15610554 PMC545968

[25] Doody HK , Peterson GM , Watson D , Castelino RL . 2015Retrospective evaluation of potentially inappropriate prescribing in hospitalized patients with renal impairment. Curr Med Res Opin. 2015;31(3):525‐535. 10.1185/03007995.2015.1010036. 25629794

[26] Alhawassi TM , Alatawi W , Alwhaibi M . Prevalence of potentially inappropriate medications use among older adults and risk factors using the 2015 American Geriatrics Society Beers criteria. BMC Geriatr. 2019;19(1):154 10.1186/s12877-019-1168-1. 31142286 PMC6542098

[27] Slugg PH , Haug MT , Pippenger CE . Ranitidine pharmacokinetics and adverse central nervous system reactions. Arch Intern Med. 1992;152:2325‐2329. 1444693

[28] Zeitoun A , Zeineddine M , Dimassi H . Stress ulcer prophylaxis guidelines: are they being implemented in Lebanese health care centers? World J Gastrointest Pharmacol Ther. 2011;2(4):27‐35. 21860840 10.4292/wjgpt.v2.i4.27 PMC3158880

[29] Sharma M , Krishnamurthy M , Snyder R , Mauro J . Reducing error in anticoagulant dosing via multidisciplinary team rounding at point of care. Clin Pract. 2017;7(2):72‐74. 10.4081/cp.2017.953. PMC5406843 28484587

[30] Thorevska N , Amoateng‐Adjepong Y , Sabahi R , et al. Anticoagulation in hospitalized patients with renal insufficiency: a comparison of bleeding rates with unfractionated heparin vs enoxaparin. Chest. 2004;125(3):856‐863. 15006942 10.1378/chest.125.3.856

[31] Ishida JH , McCulloch CE , Steinman MA , Grimes BA , Johansen KL . Gaba‐pentin and pregabalin use and association with adverse outcomes among hemodialysis patients. J Am Soc Nephrol. 2018;29(7):1970‐1978. 10.1681/asn.2018010096. 29871945 PMC6050935

[32] Kimmel PL , Fwu C‐W , Abbott KC , Eggers AW , Kline PP , Eggers PW . Opioid prescription, morbidity, and mortality in United States dialysis patients. J Am Soc Nephrol. 2017;28(12):3658‐3670. 10.1681/asn.2017010098. 28935654 PMC5698071

[33] Ishida JH , McCulloch CE , Steinman MA , Grimes BA , Johansen KL . Opioid analgesics and adverse outcomes among hemodialysis patients. Clin J Am Soc Nephrol. 2018;13(5):746‐753. 10.2215/cjn.09910917. 29674340 PMC5969477

[34] Breton G , Froissart M , Janus N , et al. Inappropriate drug use and mortality in community‐dwelling elderly with impaired kidney function–the Three‐City population‐based study. Nephrol Dial Transplant. 2011;26:2852‐2859. 21292816 10.1093/ndt/gfq827 PMC3907357

[35] Tesfaye WH , Castelino RL , Wimmer BC , Zaidi STR . Inappropriate prescribing in chronic kidney disease: a systematic review of prevalence, associated clinical outcomes and impact of interventions. Int J Clin Pract. 2017;71(7):e12960. 10.1111/ijcp.12960 28544106

[36] San‐José A , Agustí A , Vidal X , et al. Inappropriate prescribing to the oldest old patients admitted to hospital: prevalence, most frequently used medicines, and associated factors. BMC Geriatr. 2015;15(1):42 10.1186/s12877-015-0038-8. 25887546 PMC4403827

