# Supplementary material for: Potentially inappropriate medications prescribing to elderly patients with advanced chronic kidney by using 2019 American Geriatrics Society Beers Criteria
Source: Health Sci Rep. 2020 Dec 7;3(4):e214. doi: 10.1002/hsr2.214 (PMC7720279; doi:10.1002/hsr2.214)
Supplement: Supplementary file 1 — Table S1 Sixth table of the AGS Beers Criteria 2019 [file HSR2-3-e214-s001.docx]

Supplementary Table 1: Sixth table of the AGS Beers Criteria 2019

| **2019 American Geriatrics Society Beers Criteria® for Medications That Should Be Avoided or Have Their Dosage Reduced With Varying Levels of Kidney Function in Older Adults** | | | | | |
| --- | --- | --- | --- | --- | --- |
| **Medication Class**  **and Medication** | **Creatinine Clearance**  **at Which Action**  **Required, mL/min** | **Rationale** | **Recommendation** | **Quality of**  **Evidence** | **Strength of**  **Recommendation** |
| **Anti-infective** |  |  |  |  |  |
| Ciprofloxacin | <30 | Increased risk of CNS effects  (eg, seizures, confusion) and  tendon rupture | Doses used to treat common  infections typically require  reduction when CrCl  <30 mL/min | Moderate | Strong |
| Trimethoprim/sulfamethoxazole | <30 | Increased risk of worsening of  renal function and hyperkalemia | Reduce dose if CrCl  15-29 mL/min  Avoid if CrCl <15 mL/min | Moderate | Strong |
| **Cardiovascular or hemostasis** | | | | | |
| Amiloride | <30 | Increased potassium and  decreased sodium | Avoid | Moderate | Strong |
| Apixaban | <25 | Lack of evidence for efficacy  and safety in patients with a  CrCl <25 mL/min | Avoid | Moderate | Strong |
| Dabigatran | <30 | Lack of evidence for efficacy  and safety in individuals with a  CrCl <30 mL/min. Label dose  for patients with a CrCl  15-30 mL/min based on  pharmacokinetic data. | Avoid; dose adjustment advised  when CrCl >30 mL/min in the  presence of drug-drug  interactions | Moderate | Strong |
| Dofetilide | <60 | QTc prolongation and torsade  de pointes | Reduce dose if CrCl  20-59 mL/min  Avoid if CrCl <20 mL/min | Moderate | Strong |
| Edoxaban | 15-50  <15 or >95 | Lack of evidence of efficacy or  safety in patients with a CrCl  <30 mL/min | Reduce dose if CrCl  15-50 mL/min  Avoid if CrCl <15  or >95 mL/min | Moderate | Strong |
| Enoxaparin | <30 | Increased risk of bleeding | Reduce dose | Moderate | Strong |
| Fondaparinux | <30 | Increased risk of bleeding | Avoid | Moderate | Strong |
| Rivaroxaban | <50 | Lack of efficacy or safety  evidence in patients with a CrCl  <30 mL/min | Nonvalvular atrial fibrillation:  reduce dose if CrCl  15-50 mL/min; avoid if CrCl  <15 mL/min  Venous thromboembolism  treatment and for VTE  prophylaxis with hip or knee  replacement: avoid if CrCl  <30 mL/min | Moderate | Strong |
| Spironolactone | <30 | Increased potassium | Avoid | Moderate | Strong |
| Triamterene | <30 | Increased potassium and  decreased sodium | Avoid | Moderate | Strong |
| **Central nervous system and analgesics** | | | | | |
| Duloxetine | <30 | Increased gastrointestinal  adverse effects (nausea,  diarrhea) | Avoid | Moderate | Weak |
| Gabapentin | <60 | CNS adverse effects | Reduce dose | Moderate | Strong |
| Levetiracetam | ≤80 | CNS adverse effects | Reduce dose | Moderate | Strong |
| Pregabalin | <60 | CNS adverse effects | Reduce dose | Moderate | Strong |
| Tramadol | <30 | CNS adverse effects | Immediate release: reduce  dose  Extended release: avoid | Low | Weak |
| **Gastrointestinal** | | | | | |
| Cimetidine | <50 | Mental status changes | Reduce dose | Moderate | Strong |
| Famotidine | <50 | Mental status changes | Reduce dose | Moderate | Strong |
| Niizatidine | <50 | Mental status changes | Reduce dose | Moderate | Strong |
| Ranitidine | <50 | Mental status changes | Reduce dose | Moderate | Strong |
| **Hyperuricemia** | | | | | |
| Colchicine | <30 | Gastrointestinal,  neuromuscular, bone marrow  toxicity | Reduce dose; monitor for  adverse effects | Moderate | Strong |
| Probenecid | <30 | Loss of effectiveness | Avoid | Moderate | Strong |

Abbreviations: CNS, central nervous system; CrCl, creatinine clearance; QTc, corrected QT interval; VTE, venous thromboembolism.
